# Shared periodic performer movements coordinate interactions in duo improvisations

**DOI:** 10.1098/rsos.171520

**Published:** 2018-02-21

**Authors:** Tuomas Eerola, Kelly Jakubowski, Nikki Moran, Peter E. Keller, Martin Clayton

**Affiliations:** 1 Department of Music, Durham University, Durham, UK; 2 Reid School of Music, University of Edinburgh, Edinburgh, UK; 3 MARCS Institute for Brain, Behaviour and Development, Western Sydney University, Sydney, New South Wales, Australia

**Keywords:** entrainment, music, interaction, coordination, performance, wavelet

## Abstract

Human interaction involves the exchange of temporally coordinated, multimodal cues. Our work focused on interaction in the visual domain, using music performance as a case for analysis due to its temporally diverse and hierarchical structures. We made use of two improvising duo datasets—(i) performances of a jazz standard with a regular pulse and (ii) non-pulsed, free improvizations—to investigate whether human judgements of moments of interaction between co-performers are influenced by body movement coordination at multiple timescales. Bouts of interaction in the performances were manually annotated by experts and the performers’ movements were quantified using computer vision techniques. The annotated interaction bouts were then predicted using several quantitative movement and audio features. Over 80% of the interaction bouts were successfully predicted by a broadband measure of the energy of the cross-wavelet transform of the co-performers’ movements in non-pulsed duos. A more complex model, with multiple predictors that captured more specific, interacting features of the movements, was needed to explain a significant amount of variance in the pulsed duos. The methods developed here have key implications for future work on measuring visual coordination in musical ensemble performances, and can be easily adapted to other musical contexts, ensemble types and traditions.

## 1. Introduction

### 1.1. Movement coordination in verbal and non-verbal communication

Human interaction is replete with examples of the coordinated temporal exchange of information, from conversations [1] and storytelling [2] to sports [3] and dance [4]. In verbal exchanges, such coordination is necessitated due to the turn-taking nature of conversation [5], as well as the need to create shared representations and a sense of mutual understanding [6]. Behavioural manifestations of interpersonal coordination during conversation have been revealed in the form of convergence in speaking rate [7], postural mirroring and/or matching [8–10], coupling between eye movements of conversants [11] and imitation of facial expressions [12]. Such behavioural coordination can increase liking and affiliation ratings [8,13,14], elicit prosocial behaviours [15], and enhance memory for details of the conversation and conversational partner [16]. Thus, behavioural coordination serves to facilitate temporal, affective and cognitive aspects of interpersonal communication in conversation.

Musical performance is a particularly interesting instance of *non-verbal* interpersonal interaction. Many of the aspects of conversation described above, such as shared representations, a need for mutual understanding to coordinate common goals and turn-taking behaviours, are implicated in music performance [17,18]. In addition, many types of music involve high levels of synchronization between co-performers during joint musical passages, which requires precise integration of feedback between the auditory and motor systems that is developed over years of dedicated practice [19,20]. In both conversation and musical performance, interaction may take place on several timescales at once [21,22]. In music, this is particularly true since the temporal organization of the music affords the coordination of joint actions at different hierarchical levels (e.g. individual note events, rhythmic pulse/beats, phrases, higher-level structural boundaries) [23,24]. The importance of any given level may depend on aspects of the music in question, according to formal or compositional elements and performative preferences and constraints (e.g. pulsed versus non-pulsed, improvised versus scripted, modality of interaction, composition of the group, genre, instrument limitations, etc.).

Most previous research on interpersonal synchrony or entrainment in musical performance has focused on the precise temporal coordination that is needed at the level of individual notes or beats, for instance, by examining sensorimotor synchronization (e.g. tapping) with a musical stimulus (for an overview, see [25]) or note-level asynchronies or phase differences in recorded musical performances [26–32]. This study aimed to examine behavioural coordination at a higher level of the temporal hierarchy, specifically by examining relationships between larger-scale body movements of musical co-performers, such as body sway and head nods. Such movements, which are often referred to as sound-facilitating or ancillary movements, are not involved in actual sound production but serve to support the musical performance by communicating and coordinating aspects of the formal structure of a piece of music (e.g. phrase boundaries) and conveying expressive intentions either between co-performers or to an audience [26,33–40].

A more thorough account of the temporal relationships between such ancillary movements is needed, as these movements play a key role in information exchange or interaction between co-performers. Previous research has revealed that the temporal coordination of body sway between performers is systematically related to the millisecond-level synchrony that is achieved in terms of sound production [29], with evidence that body sway coordination is linked more closely to knowledge of the formal structure of the music while note-to-note synchrony is more closely related to familiarity with a co-performer’s playing style [41]. Visi *et al*. [42] presented a case study showing that the periodicities of both sound-producing and sound-facilitating movements are related to the rhythmic aspects in electric guitar playing, although this example was not extended to interpersonal coordination (e.g. duos). In addition, ancillary head movements of the same performer have been found to differ between solo and ensemble performances of the same piece [43] and between performance conditions that vary in the degree of familiarity and expressive freedom afforded by the situation [44]. Our work expanded the scope of this research area by exploring how aspects of improvising musicians’ ancillary movements contribute to observers’ perceptions of interaction, or meaningful exchange of information, between co-performers.

### 1.2. Measuring movement coordination

The collection of movement coordination and behavioural mimicry data has a long history in the conversation literature, from manual coding methods for movement types used in early studies (e.g. [45–47]) to more recently developed motion tracking technologies [10,48]. Similar measures have been taken in music research; in particular, manual coding methods have often been applied to label individual gestures [26,39,49], whereas motion capture systems are often implicated for tracking rapid movements involved in sound production, such as violin bow strokes or finger movements of pianists [28,50]. Although motion capture systems have also been used to measure ancillary movements [29,41,43], the longer timescale over which these movements occur affords the opportunity to make use of other methods. This can be advantageous, as the collection of motion capture data requires a costly and time-consuming set-up, which can be somewhat invasive and unnatural to musicians due to the need to affix markers to the body. The artificial nature of this set-up may also have adverse effects on the degree to which musicians are fully able to interact expressively with one another. The present work takes a more ecological approach, by tracking musical performers’ movements from video recordings. Video recordings are a non-invasive and inexpensive alternative that can be collected in a wide variety of real-world settings, from music festivals and gigs in nightclubs to cross-cultural field research. The work presented here makes use of automated computer vision techniques, which have been validated for use in tracking ancillary movements of musical performers from video [51]. Specifically, this method allows for the quantification of gross body movements, such as body sway and head nods, which have been implicated as key sources of co-performer interaction in previous work [34,39]. More fine-grained movements, such as smiles and eye gaze direction, may also play a role in such interactions but were beyond the scope of the present work.

A variety of analysis techniques have been applied to the types of movement data discussed above, in order to provide an index of interpersonal movement coordination (i.e. stability of the relationships between co-performers’ movements) or synchrony (i.e. exact alignment of movement events). Analysis techniques that have been used with time-series data include cross-correlation,^1^ event synchronization (ES) [53], cross-recurrence quantification (CRQ; e.g. [54]) and the cross-wavelet transform (CWT; e.g. [55]). The choice of analysis method can be informed by the data type and assumptions that need to be met for usage. For instance, ES is typically applied to binary data, where the timings of detected ‘events’ in one time series are compared to analogous events in another series, while other techniques such as CRQ and CWT are more appropriate for continuous data.

CWT analysis was selected as the most appropriate method for the present study, as we sought to explore coordination, rather than exact synchrony, using continuous movement data from improvising duos. CWT analysis examines the shared periodicities of two time series across different frequencies and time. This technique thus permits the investigation of coordination at multiple timescales, by examining movements across different frequency bands, which allowed us to make use of information from different types of ancillary movements, from fast head nods to slower swaying motions. CWT analysis has also recently been proposed as an informative tool for quantifying movement coordination between improvising musicians [24]. Specifically, Walton *et al.* [24] described different patterns of limb and head coordination using CWT analysis when piano duos were asked to improvise over different backing tracks, as well as to play in synchrony with an ostinato backing track.

### 1.3. The present study

**Figure 1.**
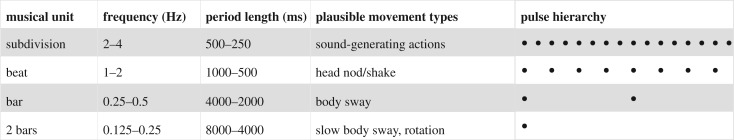
Relationship between musical units, frequency, period, plausible movement patterns and typical pulse hierarchy in metrical (pulsed) music (assuming tempo range from 60 to 120 bpm and time signature of 4/4).

The overall focus of the present work was to test whether human judgements of visually apparent bouts of interaction between co-performers in improvising duos are influenced by body movement coordination at multiple timescales. Research on musical performance can add a new perspective here beyond work on many other types of human interaction (e.g. conversation), as music offers a rich and more diverse range of temporal patterns over which it is organized, from strictly beat-based music to free improvisation across a wide range of musical tempi. Music theory describes the organization of much music as relying on the perception of a regular pulse or ‘beat’ that can be inferred from auditory information [56,57]. Beats are often assumed to fall at isochronous time intervals, although several examples are documented of non-isochronous but systematic interval sequences (e.g. [58]). ‘Metre’ describes the organization of time by two or more interacting beat levels, as for instance when every second, third or fourth beat in a sequence is felt to be stronger than the others. In figure 1, we detail how this beat structure and metrical hierarchy corresponds to movement frequencies (in hertz), and outline plausible movement types that might fit these frequencies.

The present work made use of two datasets of video recordings: one in which performances comprised a regular beat and metrical structure (standard jazz improvisations over the piece Autumn Leaves) and one in which the performance style is characterized by the avoidance of a regular, predictable beat and metre (free improvisations). This allowed us to investigate how the degree of predictability afforded by the metrical structure and pulsed versus non-pulsed nature of the music might differentially influence the types and timescales of movement coordination between co-performers.

In this work, three main research questions were outlined:

(1) How are visual bouts of interaction (as coded manually by human raters from silent videos) between musicians characterized? In particular, we aimed to describe the characteristic movement types (e.g. body parts implicated, frequency ranges of movement), duration and location within the musical form of the bouts of the interaction. We also aimed to characterize the range and commonalities between the periodicities of the movements.
(2) Can human judgements of visually apparent bouts of interaction be explained by body movement coordination at multiple timescales? Specifically, the contribution of several variables that quantified the degree of shared periodicity between the movements of the performers, movement amplitude and phase relations between the movements to predicting visual bouts of interaction were examined. As a secondary point of interest, the contribution of audio features of the music were also explored, in order to test whether auditory aspects of the performances (dynamics, pulse clarity, periodicity) explained additional variance that was not captured by the predictors derived from the visual aspects. If audio features were found to be key predictors despite the focus on the visual domain in the annotation of bouts of interactions, this could indicate that sound-producing movements, which are directly related to the audio signal, were also being interpreted as evidence of co-performer interaction. If audio features played a less prominent role in predicting visual bouts of interaction, this would suggest that sound-producing and sound-facilitating/ancillary movements serve different functions that can be differentiated by human raters.
(3) How similar are movement coordination patterns in pulsed versus non-pulsed musical performances, and what differences emerge? In particular, we aimed to investigate whether the less predictable and more diverse range of temporal structures afforded by the free improvisations resulted in greater variation in movement coordination strategies in comparison to the more strictly hierarchical metrical structure of the standard jazz performances.

These aims were explored in a first study (Experiment 1) by using quantitative measures of movement coordination to predict annotations of bouts of interaction made by four expert musicians. A second study (Experiment 2) then served to validate and expand upon several aspects of the procedures from Experiment 1, in particular by comparing predictions from the statistical models and musician annotators from Experiment 1 to ratings of visual interaction obtained from a naive sample of human participants. The outcomes of this work are important for developing computational methods that can approximate human judgements of meaningful coordination between co-performers, as the use of unsupervised (or partially unsupervised) techniques for quantifying coordinated behaviours will allow for substantial scaling-up of the amount of data that can be processed when compared with, for instance, manual coding methods. Such approaches can potentially be applied in subsequent research on video footage from entire concerts or even large corpora of video-recorded musical performances.

## 2. Experiment 1: predicting visual bouts of interaction from movement and audio features

### 2.1. Methods

#### 2.1.1. Materials

The study made use of 30 video-recorded improvising duo performances, which were created for the *Improvising Duos* corpus from the work of Moran *et al.* [59]. Fifteen of the 30 videos were of five duos performing free improvisations. This style is characterized by the avoidance of a regular, predictable beat; as such, this subset of the data is hereafter referred to as the ‘non-pulsed duos’. The other 15 were performances by six duos performing a jazz standard (Autumn Leaves). This piece has a regular underlying beat and simple metrical structure; these 15 performances will therefore be referred to as the ‘pulsed duos’. Although the musicians in the pulsed duos were free to choose their own tempo, the range of performance tempi across the different recordings in this dataset is fairly narrow (approx. 120–150 bpm [59], median of 132 bpm for pulsed duos, median of 106 bpm for non-pulsed duos, significantly different in independent-samples *t*-test, *t*_28_=2.47, *p*<0.05). Analysis of the audio data from each performance using a pulse clarity algorithm [60] revealed that the non-pulsed duos displayed lower pulse clarity values than the pulsed duos (independent-samples *t*-test, *t*_28_=4.85, *p*<0.001); the non-pulsed duos also exhibited fewer audio events per second on average than the pulsed duos (*t*_28_=4.27, *p*<0.001; see table 1 for full comparison of the two datasets).

**Table 1.** Summary of pulsed and non-pulsed duo datasets (videos, annotations of interaction and musical structure, and audio data descriptors). Durations are in seconds. The audio data descriptors, event density, tempo and pulse clarity are summarized with median values. Pulse clarity ranges from 0 to 1 where 1 is the clearest possible pulse sensation. These three descriptors were determined with MIR Toolbox [62] using default parameters.

		pulsed duos		non-pulsed duos	
		count	median dur. (s.d.)	count	median dur. (s.d.)
video	duration of videos	15	114.2 (25.8)	15	177.6 (63.3)
	number of duos	6	n.a.	5	n.a.
	instruments	6	n.a.	6	n.a.
annotation	interactions	97	3.8 (5.1)	63	10.9 (16.0)
	upper torso	59	6.4 (4.2)	48	9.3 (15.2)
	head	36	3.6 (6.9)	8	13.2 (15.5)
	other	2	3.9 (3.7)	7	21.5 (20.7)
structure	joint sections	37	47.7 (12.4)	40	40.0 (38.2)
	solo sections	60	7.7 (12.9)	23	26.2 (16.2)
audio	audio event density		1.12 s^−1^ (0.56 s^−1^)		0.45 s^−1^ (0.29 s^−1^)
	tempo		132 bpm (32)		106 bpm (18)
	pulse clarity		0.156 (0.059)		0.065 (0.034)

Each duo contributed two to three video recordings to the dataset. Video recording duration ranged from 98.3 to 336.5 s (*M*=157.0, *s*.*d*.=55.7). The duos comprised a variety of instruments, including saxophone, piano, double bass, electric bass, drums, trumpet, guitar, flute, clarinet, violin and cello. Performers in these duos were recruited on the basis of public performance experience of around 10 years in their respective styles. Some performers had played together before, but this was not a primary recruitment criterion. No performer played in more than one duo. In all duos, performers were situated in a similar position—in which both performers could see one another—and at a similar distance from the camera, having been advised by research team to face one another, and encouraged to perform in a standing position with their feet within a prescribed zone. For more information, these videos are available at http://datashare.is.ed.ac.uk/handle/10283/2840. All videos were recorded in the same room under similar performance conditions at the Max Planck Institute for Human Cognitive and Brain Sciences in Leipzig, Germany using a Sony HDR-HC9 camera at 25 Hz. Audio was recorded at a sampling rate of 48 kHz to a separate audio track for each musician using two Audio Technica AT 2035 condenser microphones; in addition, digital instruments were recorded with a direct line in. However, performers were placed very close to each other, and therefore there was a significant amount of spill from the other instrument on most audio tracks.

#### 2.1.2. Annotation of the video datasets

Manual annotation of interaction between the performers was completed in ELAN [61] by four expert musicians (authors N.M., K.J., T.E. and M.C.), with each video recording being coded by three of the four annotators. Annotators watched all videos with the audio muted, as the task was to code perceived bouts of visual interaction between performers without being influenced by audio cues. Annotators first watched each video in its entirety without making annotations, in order to familiarize themselves with each duo’s typical movement qualities before coding ‘bouts of interaction’. Such bouts were defined as periods of interaction arising from the behaviour of the performers, where the characteristic movement patterns of the two musicians indicated a degree of correspondence in the eyes of the annotator. Annotators were instructed to make use of terms such as ‘matching’, ‘corresponding’ or ’complementing’ in briefly describing the event, and to make note of any specific body parts that influenced their decision to code a bout of interaction. For example, one particular bout of interaction in the dataset was labelled by three separate annotators as ‘complementary nodding/leaning’, ‘intermittent coordination between slow body sway’ and ‘similar slow sways at the moment’.

This process produced 455 annotated bouts of interaction in total, with a median of five annotations per annotator for each video. The annotated segments varied in duration from 0.74 to 75.4 s (*M*=10.9, *s*.*d*.=11.6). In total, 72.5% of the annotation time series from different annotators overlapped. Consistency between the annotators was explored by calculating the overall interrater agreement across the three annotators. First, each pair of annotation time series was matched through dynamic time warping using a constrained, asymmetric window to adjust the small timing discrepancies between the annotators. These time-adjusted data were subjected to the analysis of interrater agreement, which resulted in an average *κ* (Cohen’s Kappa) of 0.797 (*Z*=13.8, *p*<0.001) across all 90 pairings (30 videos, three pairings for each). This result suggested a reasonable degree of interrater agreement, despite some variation in the annotations.

For the subsequent analyses, we formed an aggregate of the original, individual raters’ annotations by identifying a bout of interaction as any time segment when at least two annotators had marked the existence of interaction. This operation mitigates the small timing inaccuracies between annotators and eliminates the bouts of interaction only proposed by a single annotator. After this operation, 160 bouts of interaction remained, which typically lasted for about 6 s (see table 1 for complete descriptions).

In addition to the annotation of bouts of interaction, the musical structure of all 30 video recordings was labelled by one of the annotators, with each section of the recording classified as either joint performance (77 instances across the 30 videos) or solo sections (83 instances). Solos tended to be shorter than the joint sections (table 1); this was particularly the case in the pulsed duos, because the performers typically took turns soloing over short sections of the piece (‘trading solos’, often over eight or four bars).

Finally, the qualitative descriptions provided by the annotators within the 160 aggregated bouts of interactions were coded by one of the annotators in terms of the key body part(s) of the performers that were implicated in each bout. This revealed that upper body/torso movements dominated (73.0%) over head movements (23.9%), and any other body part (foot, hand, eye contact, etc.) provided a relatively rare (3.1%) source of interaction (table 1).

#### 2.1.3. Movement extraction

Automated movement quantification was implemented using dense optical flow (OF) estimation in EyesWeb XMI 5.7.0.0.^2^ OF is a standard computer vision technique that performs two-dimensional movement tracking on video data by estimating the apparent velocities of objects. The EyesWeb implementation of OF that was used in this study is based on the algorithm of Farnebäck [63] and has been validated for use in movement tracking in music performance using a diverse range of video-recorded materials (with different camera angles, instruments, performer positions and clothing) in Jakubowski *et al.* [51]; for an application of OF in studying movement coordination in conversation see also [14]. For each video, two regions of interest (ROIs) were manually selected that corresponded to the upper body region of each performer. OF tracking was then applied to each ROI, resulting in a series of *x*- and *y*-coordinates for the barycentre of the ROI for each frame of the video, where the video sampling rate was 25 Hz. To compute the barycentre coordinates, the image is converted to greyscale and the coordinates are calculated as a weighted mean of the pixel intensities within the ROI. The ROIs were constrained to the upper body because the manual annotations revealed that the head and upper body contributed to the vast majority of interactions (96.9%). The *x*- and *y*-coordinates of the performers’ movements were smoothed using a Savitzky–Golay filter with an order of three and length of five frames set heuristically to remove noise inherent in the OF output. The *x*- and *y*-coordinates were then converted into polar coordinates and reduced into radial coordinates (*ρ*), which were also detrended and normalized to a range of 0–1 for the analysis. In addition, an overall quantity of motion estimate was extracted from each ROI using a frame differencing (FD) method in EyesWeb. The implementation of FD was based on the Pfinder algorithm of Wren *et al.* [64], in which adaptive background subtraction is performed while calculating pixel change from frame to frame on the foreground element(s), in this case the upper body of each performer within each ROI (see [51] for further details).

#### 2.1.4. Audio extraction

**Figure 2.**
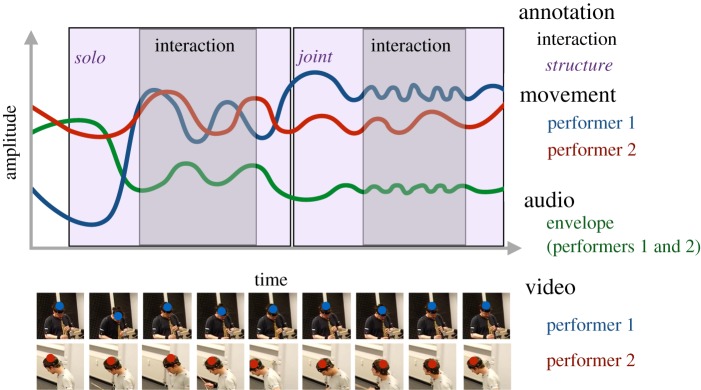
Example visualization of the data (video, annotation, movement (combined *x* and *y*) and audio). The blocks display the manual annotations (two bouts of interaction are shown in grey and musical structure is denoted in purple). The red and blue lines display the raw amplitude of the movements for two performers. These are the radial coordinates of the *x*- and *y*-coordinate positions obtained from the optical flow analyses of the videos, with the video ROIs and the detected centres of the movement shown in the lower pane. The green line represents the amplitude of the audio envelope.

To capture the auditory aspect of the performances, the envelope of the combined performance (performers 1 and 2) was extracted from the videos. As the performances consisted of diverse instrument combinations, there was no uniform recording set-up. For this reason, audio separation was not attained for each individual instrument, because such recordings would have created constraints that impaired communication between the improvisers (typically, musicians would play in sound-proofed booths if separation is needed). Nevertheless, the joint audio signal provides a continuous rather than discrete characterization of the note onsets and dynamics within the performances. This representation of the audio also does not attempt to define discrete onsets for cases in which they are virtually impossible to detect, that is, for smooth glides or long sustained sounds that are present in some of the performances in these datasets. The envelopes were extracted from the audio data with MIR toolbox 1.6.2 using a 100 Hz sampling rate and summing the half-wave rectified envelope with the non-differentiated envelope (*λ*=0.1) and Gaussian smoothing (*σ*=3) [65]. Figure 2 summarizes the extracted data, including the manual annotations, and movement and audio data.

#### 2.1.5. Wavelet transformation

The CWT is a sophisticated method for analysing relationships between periodicities within time series [55,66]. This method originated within the biological sciences but has recently found applications within psychology, particularly for studying the temporal dynamics of human interaction [67]. In essence, CWT analysis characterizes the relationships between wavelet transforms of two separate time series. It can help to disentangle the relationships between the two time series in terms of time and frequency. The advantages of CWT analysis over other methods (discrete relative phase, Hilbert transform, cross-correlation, fast Fourier transform) are that it is able to handle frequencies at different timescales and extract the phase information accurately in such situations. The underlying operations of CWT analysis are based on the wavelet transform (WT) [68], which describes the local properties of a time series using scalable, discrete wave functions (wavelets). The temporal resolution of the WT method is dynamic and adjusts itself to different frequencies, offering an excellent trade-off between the time and frequency domains [69]. CWT analysis is an extension of the WT in which the CWT of the signal is the pointwise multiplication of two wavelet-transformed signals. This yields the energy of the interaction between the two time series across the frequency ranges specified and across time. In addition, the analysis offers relative phase information within the specified frequency bands and time. This is a flexible technique that tolerates changes in frequency, amplitude and the combination of both.

**Figure 3.**
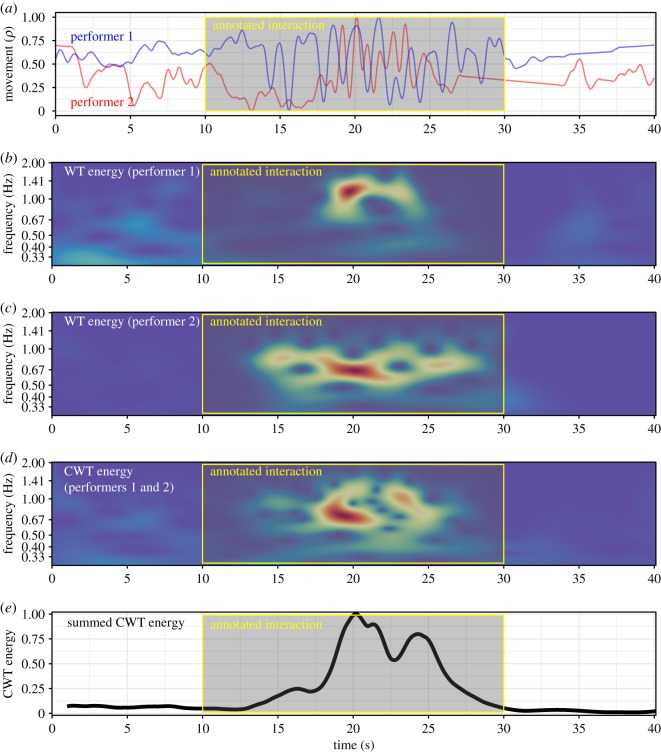
CWT example from the data (non-pulsed, ID Free_VP0506_08 at the dataset, extract from 50 to 90 s). Panel (*a*) shows the combined movement of each performer, panels (*b*) and (*c*) display the individual WT for each performer, panel (*d*) exhibits the CWT and panel (*e*) shows the energy of the CWT.

In this study, we first examined the movement frequencies of the individual performers using WT analysis and then explored the relationship between the movements within each duo using CWT analysis. To capture the potentially wide range of variations in movements within the datasets, the CWT analysis was applied across a broad frequency range (0.3–2.0 Hz) to each duo’s movement data, and measures of CWT energy and phase were extracted from this analysis. A visualization of the process in given in figure 3.

For the audio data, the energy of the signal (root mean square of the envelope sampled at 100 Hz) was first extracted to describe the overall dynamics of the musical performance. Using this variable, the periodicity of the signal was estimated using a WT within the frequency range from 0.25 to 10 Hz. A wider frequency range was used for the audio data analysis than the movement data due to the higher sampling rate at which the audio data were collected as well as the faster periodicities over which audio events are likely to occur than ancillary movements (the fastest subdivision of a musical beat is estimated at 100 ms, or 10 Hz [57]). All wavelet analyses were carried out using the WaveletComp package in R [70] with Bartlett windowing.

### 2.2. Results

#### 2.2.1. Characterization of performers’ behaviours

**Figure 4.**
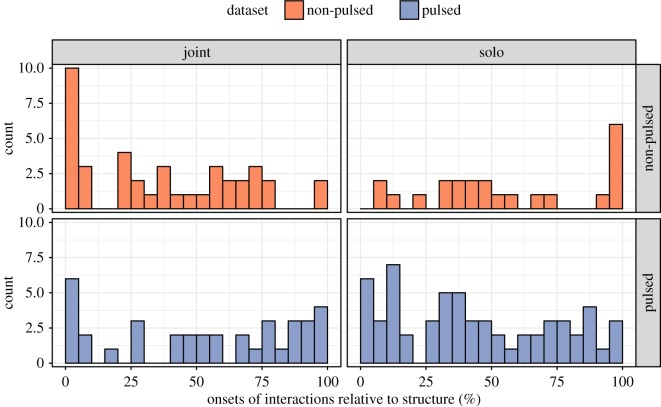
Onsets of annotated bouts of interaction across music structural sections and datasets. The *x*-axis shows the relative onset of each structural section (0–100%), and the *y*-axis indicates the frequency of the onsets of the annotated bouts of interaction.

We first examined the onsets of the annotated bouts of interaction relative to the annotated music structural sections (i.e. whether performers were playing together (joint sections) or soloing (solo sections)). One plausible function of the bouts of interaction could be to coordinate the transition involved in switching from one solo to another or between solo and joint sections (cf. [71]). To investigate this question, onsets of the bouts of interactions were binned into categories of 5% in width in terms of their relative position within the music structural sections (figure 4). There were some indicators that interactions often commenced near structural boundaries; the distribution of onsets was different between the two datasets in a chi-square test (non-pulsed versus pulsed duos; *χ*^2^=67.9, *p*<0.05), but overall, no significant difference was found in relation to joint versus solo sections (*χ*^2^=57.7, *p*=0.08).

As shown in figure 4, it was twice as likely that an onset of a bout of interaction was located within the first or last 5% of the structural section in non-pulsed duos in comparison to pulsed duos. The uneven distribution in the non-pulsed duos is understandable due to the need to coordinate moments of transition between musical sections. In the pulsed duos, the lack of such boundary signalling via visual cues may reflect the fact that the improvisers performed within a familiar, beat-based musical structure. It seems in this case that this so-called ‘trading solos’, where performers alternate between playing together and each taking their turn in performing a solo (see [59] for details), does not put particular emphasis on coordinating performances via visual interactions near section boundaries. Instead, such transitions may be coordinated by auditory cues, including both timing and tonal cues (see Hadley *et al*. [72], who found that tonal cues allow listeners to more accurately predict the end of a solo in standard jazz improvisation than free improvisation).

#### 2.2.2. Describing performers’ movements and audio data

To diagnose the overall periodic patterns exhibited by the movement and audio data from both datasets, we first examined an overview of the wavelet-transformed signals obtained from the movements of the individual performers and the envelope of the combined audio data. These descriptive analyses were confined to the frequency range from 0.30 to 2.0 Hz; this choice of frequency range was dictated by the typical movement frequencies exhibited in music performances [28,73] and the type of data available (movements extracted from videos recorded at 25 Hz). The range also roughly corresponds to movement frequencies at the level of individual musical beats to several bars of music (figure 1).

**Figure 5.**
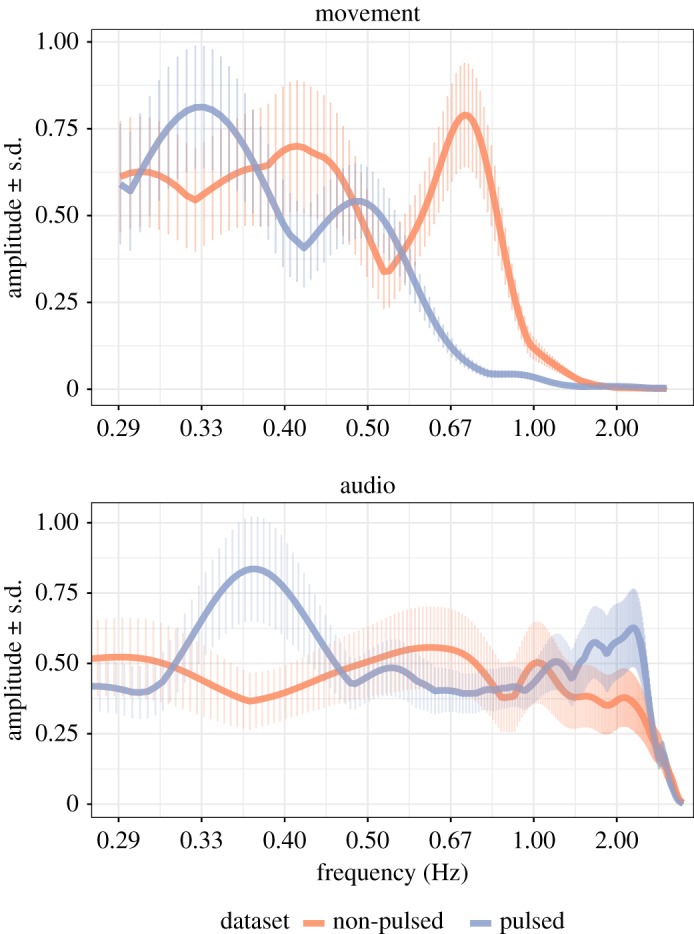
Wavelet amplitudes (means and standard deviations) for movement and audio aggregated across all performers.

The wavelet analysis results from the movement data, summarized in figure 5, demonstrate a broad range of periodic behaviours with frequency peaks that vary considerably across the datasets. Both datasets display two frequency peaks: for the non-pulsed duos these are at 0.75 and 0.40 Hz, whereas in the pulsed duos the peaks occur at lower frequencies (0.50 and 0.33 Hz). It is interesting that the period lengths corresponding to each of these pairs of frequency peaks are approximately 1 s apart, possibly reflecting broad movement types such as head nods (faster frequencies) and body sway (slower frequencies), which would be consistent with past research on dance [74] and gestural communication in duo performances [29,75,76].

The audio data revealed a tendency towards faster frequencies in the pulsed duos in comparison to the non-pulsed duos, despite the opposite trend in the movement data. This naturally reflects the beat-driven nature of the moderate-tempo jazz standard that the pulsed duos performed (Autumn Leaves) that displayed faster tempi (132 bpm, which roughly corresponds to the right-most peak at 2.18 Hz in figure 5) than the non-pulsed duos (106 bpm, see table 1). Also of note is the peak around 0.375 Hz in the audio data for the pulsed duos, which could potentially relate to phrase patterns (a period of 2.67 s is approximately half the solo phrase duration, which is typically around 5.3 s long).

**Figure 6.**
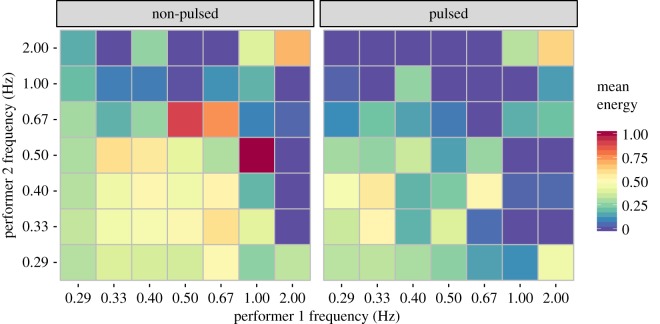
Co-occurring movement frequencies across the datasets. The axis labels denote the centre frequencies of the non-overlapping frequency bands used in the analysis.

What the previous summaries do not reveal is the *simultaneous* periodic activity of both performers in each duo. Figure 6 visualizes the patterns of co-occurrent movement frequencies between performers. Specifically, the energy of the maximum frequency across seven non-overlapping frequency bands (centred around 0.29, 0.33, 0.40, 0.50, 0.67, 1.00 and 2.00 Hz) in each frame for both performers is collapsed across time. These normalized two-dimensional density plots portray similar overall patterns to the individually aggregated frequency summaries (figure 5), as the non-pulsed duos tend to display faster movements than the pulsed duos. More importantly, identical movement frequencies of both performers are relatively uncommon in both datasets. In the non-pulsed duos, identical shared movement frequencies occur around 0.67 and 2.00 Hz, but there are stronger relationships between non-identical frequencies such as 0.50 Hz co-occurring with 0.67 Hz and 1.00 Hz co-occurring with 0.50 Hz. In the pulsed duos, both performers occasionally sway at the same frequency, mainly at low frequencies (0.33 Hz) or high frequencies (2.0 Hz). There are also various non-matching pairings at other frequencies such as 0.33 and 0.40 Hz, as well as 0.40 and 0.67 Hz.

To recapitulate, the analysis thus far has described the range of movement frequencies and their mutual relationships, as well as the periodicities of the music (audio) itself. These descriptive results helped us to focus the subsequent analysis of interaction on plausible aspects of behaviours.

#### 2.2.3. Prediction of interaction bouts with movement and audio variables

The primary aim of the subsequent analysis was to quantify the degree to which measures of co-occurrent movements of the pairs of performers could be used to predict visually apparent bouts of interaction, as coded by the annotators. In the initial stage of this analysis, we defined nine potential movement predictors, which are outlined in table 2. As the summaries in figure 5 revealed a broad range of different movement frequencies within the datasets, the CWT energy measure across the broad frequency band from 0.3 to 2.0 Hz was included as a first predictor of interest (Movement CWT Energy (Broad)). The phase information from this broadband CWT analysis was also included (Movement CWT Phase). As the descriptive results also revealed some peaks in the data indicating that certain movement frequency ranges were more prevalent than others, we applied the CWT analysis to a more specific set of frequency bands in order to further deconstruct the prominent time–frequency regions relevant for the performers’ behaviours. Five non-overlapping frequency bands were defined, which were 0.3, 0.4, 0.6, 0.9 and 2.0 Hz.^3^ To capture moments where both performers were moving in a periodic fashion but these periods were unrelated (as apparent in several unrelated frequencies in figure 6), the WT for each individual performer was computed across the frequency range from 0.3 to 2.0 Hz and the WT energy of both performers was summed (Movement WT Energy (Any)). Finally, the quantity of motion—extracted with the frame differencing method from each ROI, as described in §2.1.3—was summed across the two performers in each duo and taken as an index of the overall amount of movement (Movement Quantity).

**Table 2.** Predictors of annotated bouts of interaction (movement and audio predictors).

	predictor name	description
1	Movement CWT Energy (Broad)	energy of the cross-wavelet transform, computed using both performers’ movements over a broad frequency band (0.3–2.0 Hz)
2	Movement CWT Phase	phase of the cross-wavelet transform, indicating the momentary lead/lag relationship between the performers
3–7	Movement CWT Energy (CF)	energy of the cross-wavelet transform, computed using both performers’ movements over narrow frequency bands where the centre frequencies (CF) were 0.3, 0.4, 0.6, 0.9 and 2.0 Hz
8	Movement WT Energy (Any)	energy of the wavelet transform, computed for each individual performer and summed within each duo (0.3–2.0 Hz), representing the momentary amount of periodic movement
9	Movement Quantity	summed quantity of motion from both performers, computed using frame differencing
10	Audio RMS	amplitude envelope of the audio signal in terms of the root mean square energy
11	Audio WT Energy (Broad)	energy of the wavelet transform, computed from the audio envelope over a broad frequency band (0.25–10 Hz)
12	Audio Pulse Clarity	clarity of the pulse sensation, computed using a computational model relying on periodicities assessed from the audio envelope [60]

As a secondary point of interest, we included three predictors computed from the audio data of the duo performances, to test whether some auditory aspects of the performance (e.g. sound-producing movements) influenced the judgements of bouts of interaction, despite the purely visual nature of the annotation task. The audio predictors were the root mean square energy of the audio envelope (Audio RMS), the WT energy computed from the Audio RMS variable across a broad frequency band from 0.25 to 10 Hz (Audio WT Energy (Broad)) and the clarity of the pulse sensation (Audio Pulse Clarity), which was computed with an existing model of pulse clarity [60] (table 2).

The 12 predictors were extracted across the 30 performances and downsampled to match the video frame rate (25 Hz). For all analyses, we eliminated the first and the last 5 s of each performance to avoid artefacts from the annotations and CWT analysis. This left us with 110 250 video frames (73 min and 3 s), which we downsampled by a factor of 5 for the analysis. The resulting 22 050 observations (13 161 in the non-pulsed duos; 8889 in the pulsed duos) were converted into *z*-scores for the analyses. No outliers were removed because we made use of classification models that are tolerant to violations of normality.

The analysis was completed in two stages. In the first stage, we identified the classification accuracy of all 12 predictors and trimmed the incidental ones. In the second stage, we assessed the classification accuracy of the remaining set of predictors using two complementary classification techniques: logistic regression and random forest classification. Logistic regression is an efficient and commonly applied technique for modelling the relationships between predictors and classes. The downside is that discovering potential interactions and nonlinear relationships between the predictors requires explicit postulation of such operations that is not feasible in explorative research. The random forest technique offers the benefits of decision trees, which are able to capitalize on interactions between the predictors and have good tolerance for noise and outliers [77,78]. The first stage of the analysis and initial part of the second stage were carried out using a training set—a random sample of 80% of the data—and the remaining 20% was used for the model evaluation stage. Within the training set, we carried out 10-fold cross-validation with 10 repeats to avoid overfitting. Following the second stage of the analysis, the individual contributions of the predictors from the most parsimonious models were investigated in more detail.

#### 2.2.4. Variable selection, model fitting and evaluation

**Figure 7.**
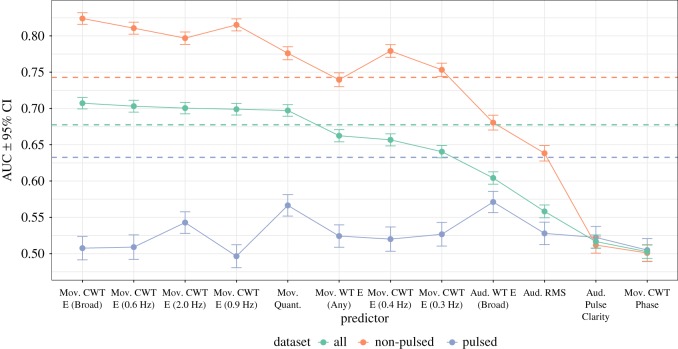
Prediction rates of bouts of interaction for each predictor across and over the datasets. Mov. denotes movement predictors and Aud. denotes audio predictors (see table 2 for details). CWT denotes cross-wavelet transform, WT denotes wavelet transform and E denotes Energy. Dotted horizontal lines indicate the baseline-level prediction for each dataset.

We first assessed how well each of the 12 predictors was able to classify each frame in the sequence in terms of the interaction class (interaction/no interaction) using logistic regression. We used the area under the curve (AUC) from the receiver operating characteristic curve as an index of classification performance, because it offers a robust scheme against overfitting, especially when the observations are unevenly distributed [79]. This analysis was performed separately for the pulsed duos, non-pulsed duos and the combined dataset (all duos) using the training dataset. The results are summarized in figure 7.

Figure 7 displays the prediction rates of annotated interactions with each feature alone, organized in descending order based on prediction rates across both datasets. This summary suggests that at least four of the predictors failed to predict interaction bouts in any of the datasets—specifically, Movement CWT Phase, Audio Pulse Clarity, Audio RMS, and Audio WT Energy (Broad). It is worth noting that the two interaction classes (interaction/no interaction) were not evenly distributed (interactions were coded for 37% of observations in the non-pulsed duos, 26% of observations in pulsed duos and 32% of the combined dataset), and thus the classification rates needed to reach levels well beyond 0.50 in order to differ significantly from the baseline rate that always predicts the majority class (no interaction). Another notable result from this analysis is that there was a marked difference in prediction accuracies between the non-pulsed and pulsed duos; in the pulsed duos, the annotated interaction bouts were generally difficult to classify with the individual predictors, but several movement features substantially predicted interactions in the non-pulsed duos.

For the second stage of analysis, we formulated two feature sets based on this initial screening stage. First, we eliminated the four lowest performing predictors that failed to predict the interactions in any dataset (Audio Pulse Clarity, Movement CWT Phase, Audio RMS and Audio WT Energy (Broad)), leaving us with eight features. Second, we compiled a feature set comprising only the five features that could predict interactions in the combined dataset (the green line denotes the baseline level for the combined dataset in figure 7). These five features were: Movement CWT Energy (Broad), Movement CWT Energy at 0.6, 2.0 and 0.9 Hz, and Movement Quantity. These two sets of predictors (eight features and five features) were entered into the logistic regression and random forest classification models with 10-fold cross-validation. The random forest model parameters were fixed, with the number of trees set to 500 and the number of variables to include in the tree model to the square root of the number of variables in the model. Finally, we added a 1-predictor model for comparison, which included only the strongest predictor (Movement CWT Energy (Broad)) from the screening stage. The model predictions were assessed with the evaluation subset of the data.

The results of the models obtained with the training sets as applied to the separate evaluation sets are displayed in table 3, including the AUC and 95% confidence intervals. Barring a few exceptions (the 1-predictor models and logistic regression models for the pulsed duos), most models reached satisfactory to excellent classification rates in all analyses using 1, 5 or 8 features. Logistic regression models displayed good prediction rates for the non-pulsed duos and combined dataset with only a single predictor, but these models surprisingly failed to deliver statistically significant improvements when additional predictors were added. This suggests that there were either interactions between the predictors that we failed to specify in the models, or that there were nonlinear relationships between the predictors and classes. If interaction terms are added to the logistic regression models, the classification rate increase is statistically significant for the 5-predictor model for the combined dataset (AUC increases from 0.731 to 0.742 with 26 additional variable combinations), but no improvement is seen for the 8-predictor model with the addition of rather numerous (191) interaction terms.

**Table 3.** Classification (AUC and CI_95_) rates across datasets and feature sets.

		AUC (CI_95_)	AUC (CI_95_)	AUC(CI_95_)
	dataset	1 predictor	5 predictors	8 predictors
logistic	non-pulsed	0.838 (0.822–0.853)^***^	0.845 (0.830–0.860)	0.850 (0.835-0.865)
	pulsed	0.510 (0.478–0.542)	0.585 (0.554–0.616)	0.590 (0.560–0.620)
	all	0.719 (0.703–0.734)^***^	0.731 (0.716–0.747)	0.731 (0.716–0.747)
random forest	non-pulsed	0.768 (0.750–0.786)^***^	0.942 (0.934–0.951)^***^	0.984 (0.981–0.988)^***^
	pulsed	0.493 (0.462–0.524)	0.878 (0.859–0.897)^***^	0.968 (0.959–0.977)^***^
	all	0.620 (0.603–0.637)	0.884 (0.874–0.895)^***^	0.967 (0.961–0.972)^***^

*p<0.05, **p<0.01, ***p<0.001 for leftward comparisons of AUCs with DeLong’s test using predictions on the evaluation sets by the models from the training sets.

A more elegant way of capturing both interactions and nonlinear patterns within the data is to use random forest models. These models are not strictly comparable to logistic regression for a 1-predictor model, in which they exhibited poor performance, because random forests are intended for use with multiple predictors. With added predictors, however, the classification rates improved significantly (*p*<0.001 with DeLong’s test between ROC curves of the models), reaching good prediction rates for the 5-predictor models (*AUC*=0.884 for the combined dataset) and excellent rates for the 8-predictor models (*AUC*=0.967 for combined dataset). These results indicate that random forest models offer significant improvements in classification performance for these datasets, although with the trade-off of increased model complexity. In addition, the classification errors were unevenly distributed for most models. For the 1-predictor logistic regression model using the combined dataset, the confusion matrix indicates that 4.8% of the annotated interactions were misclassified by the model (false negatives), whereas 26.3% of observations were predicted as interactions when they were not annotated as such (false positives). Similar asymmetric errors were revealed in other models using the combined dataset (5.4% false negatives versus 12.8% false positives for the 5-predictor random forest model), which probably reflect the unbalanced distribution of annotated interactions (32.2%) and non-interactions (67.8%).

In sum, the annotated bouts of interaction were successfully predicted, primarily by relying on the CWT energy of the movements across a broad frequency range (1-predictor model). To gain a more precise understanding of the predictor contributions to the more complex models, we computed the variable importance of each predictor from the 8-predictor random forest models and examined the decision tree splits as a way of characterizing the interactions between the predictors.

#### 2.2.5. Contribution of the predictors

**Figure 8.**
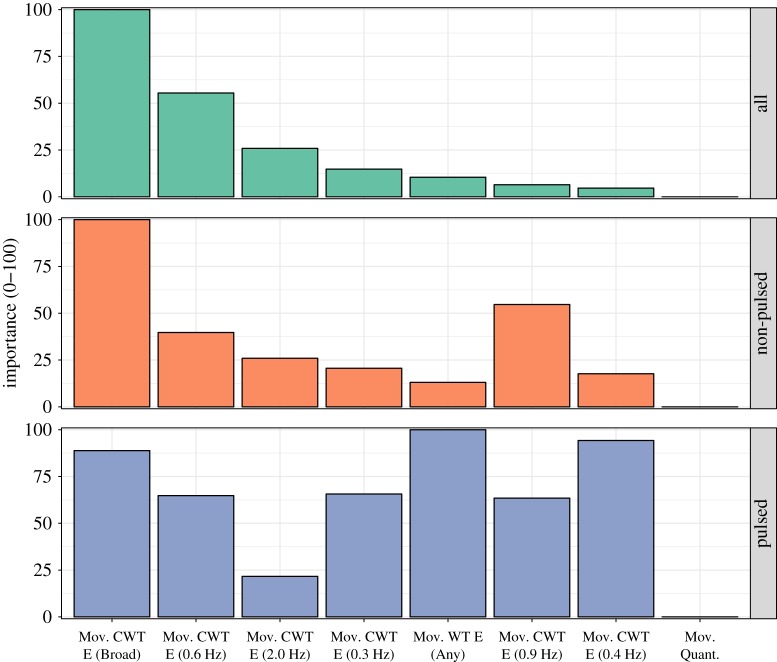
Normalized predictor importance values for the eight predictors in the random forest model across the datasets.

To explore the contributions of the individual predictors to the models, we focused on the random forest method, because this method makes use of more sophisticated techniques than logistic regression for assessing the relative importance of the predictors. Specifically, random forest models make use of the mean decrease in the contribution to the classification rate, by calculating the decrease in prediction accuracy when the variable of interest is left out of the analysis using the out-of-bag data. This technique has repeatedly performed better than other candidates in large-scale simulation studies (e.g. [80]). We describe the tree models for each dataset separately in the electronic supplementary material, S1, and here focus on the normalized predictor importance across the datasets, as displayed in figure 8.

The overall pattern of feature contributions presented in figure 8 is generally consistent with the results of the initial variable selection stage; the same three predictors—Movement CWT Energy (Broad), Movement CWT Energy (0.6 Hz) and Movement CWT Energy (2.0 Hz)—that made the greatest individual contributions in the first analysis (shown in figure 7) were also the most important predictors in the random forest. However, further down the list there are some notable differences between the initial, individual predictor analysis and the results from the 8-predictor random forest model. For instance, the Movement CWT Energy (0.4 Hz) and Movement Quantity predictors performed well individually, but did not make notable contributions in the random forest model for the combined datasets, perhaps due to collinearity with other predictors (e.g. *r*=0.47 between Movement Quantity and Movement CWT Energy (0.4 Hz)). A comparison of the predictor importance values between the pulsed and non-pulsed duos also reveals that these datasets differ markedly in terms of the key features implicated in the models, except that Movement Quantity is not a substantial predictor in either model. The interaction bouts in non-pulsed duo performances were best predicted by the cross-wavelet energy of the movements across a broad frequency range, followed by moderately fast co-occurring movements, as indexed by Movement CWT Energy in frequency bands centred around 0.9 Hz and 0.6 Hz. A different pattern emerged in the pulsed duo performances, in which interactions were characterized by slower shared periodic movements (Movement CWT Energy (0.4 Hz)) and periodic movements at unrelated frequencies (Movement WT Energy (Any)). These differences between the datasets are consistent with the patterns previously observed in the descriptive analyses (figures 5 and 6).

### 2.3. Discussion

To summarize, bouts identified as interactive within the non-pulsed duo performances were characterized by shared periodic movements across a broad frequency range, although there was also some tendency for mid-range-frequency movements to be more indicative of interactions than fast- or slow-frequency movements. In the pulsed duo performances, slow swaying movements tended to characterize the interactions, and fast periodic movements (head nods, etc.)—although relatively common in this dataset—were less indicative of interactions. It may be that these faster periodic movements are instead embodied expressions of pulse sensation, which do not necessarily serve a communicative function between co-performers.

Shared periodic movement of the performers within a broad frequency range (0.3–2.0 Hz) was the single most important predictor of interactions and delivered a satisfactory classification rate when the two datasets were combined (*AUC*=0.719). The 1-predictor classification rate was excellent in the non-pulsed duo dataset (AUC = 0.838), but poor in the pulsed duo dataset (*AUC*=0.510). Adding cross-wavelet energy measures within more specific frequency bands, the overall quantity of movement and a measure of the overall periodic movement without reference to joint periodicity did improve the classification rates across the datasets, but only when we modelled the relationships between the predictors with random forest models that are able to capture the interactions between the predictors and nonlinear patterns in the data. Simple additive logistic regression models failed to improve the model classification rates.

These results provide a first indication that computational measures of joint movement can serve as an index of co-performer interaction in the visual domain. To further validate this method and probe the errors that the statistical models are susceptible to, we ran a second experiment in which we obtained ratings of co-performer interaction for a subset of the video data from Experiment 1 from an independent sample of participants.

## 3. Experiment 2: validation of methods and models for measuring visual aspects of co-performer interaction

### 3.1. Aims

In Experiment 2, we sought to validate the results and to understand further the potential limitations posed by the methods and modelling procedures implicated in Experiment 1. Specifically, we collected ratings of co-performer interaction using a subset of the duo recordings from Experiment 1 from a sample of naive participants. The aims of this work were to assess:

(1) Whether interaction ratings obtained from a new sample of participants who were unaware of the purpose of the experiment were consistent with ratings obtained from the four musician annotators in Experiment 1. This was an important check, as the Experiment 1 annotators—who were among the authors of this paper—had some prior familiarity with both the visual and audio aspects of the video-recorded performances, and it is possible that they had certain unconscious biases in the interaction annotation task due to the fact that they were informed of the research aims.
(2) How the classification successes and errors of the simplest (1-predictor logistic regression: Movement CWT Energy (Broad)) computational model from Experiment 1 related to interaction ratings obtained from the new participant sample.
(3) Whether classification differences between the annotators and computational models in Experiment 1 could be due to the different amounts of visual information afforded to each. Specifically, the annotators had viewed the full videos, whereas the computational analysis was performed using movement data that were obtained from cropped versions of the videos, using constrained ROIs set around the upper body of each performer.
(4) Whether participant ratings of co-performer interaction differed based on the musical style, by comparing data from the pulsed and non-pulsed duos datasets.

In sum, we explored the degree to which participants’ judgements of interaction aligned with the annotators and computational model from Experiment 1 and whether these results varied as a function of amount of visual information (full/cropped videos) and dataset (pulsed/non-pulsed duos).

### 3.2. Methods

#### 3.2.1. Materials and stimuli

We selected 48 7.5-s excerpts from the video corpus that represented successes and failures of the 1-predictor model (Movement CWT Energy (Broad)) in predicting the manual annotations of interaction, while controlling for quantity of movement. Twelve excerpts represented each of the four possible model prediction categories (true positives, true negatives, false positives and false negatives). The predicted cut-off value for the 1-predictor model was established with the Youden index [81], and was calculated using the data from both the pulsed and non-pulsed duos. To select excerpts that spanned a wide range of values in terms of quantity of motion that also were matched across the four prediction categories in terms of this variable, half (6) of the selected excerpts in each of these categories were chosen to represent high overall movement amplitude and half were selected to represent low overall movement. Specifically, Quantity of Motion estimates (computed in the same way as the Movement Quantity variable in Experiment 1, using frame differencing applied to the upper body ROI of each performer) from each video excerpt were summed across the two performers and split into high/low categories using a median split. Each of the three criteria (annotation class, 1-predictor model prediction, Movement Quantity) had to be fulfilled in the majority of sequence (at least 4 s out of 7.5 s) to be included. In total, 22 of the 48 selected video excerpts were drawn from the pulsed duos dataset and 26 were from the non-pulsed duos. Following the stimulus selection procedure, there were no significant differences in Movement Quantity in independent-samples *t*-tests between the four stimulus categories (true positives, true negatives, etc.; all *p*>0.05) or the two datasets (pulsed/non-pulsed, *t*_58_=0.708, *p*=0.482).

Finally, two versions of each of the 48 excerpts were produced. One version made use of the full visual information from each video excerpt, while the second version was cropped by making visible only the sections of the video (upper bodies of the two performers) that had been selected as ROIs for the optical flow movement tracking procedure. The median ROI size was 158×158 pixels, which is approximately one-fourth of the original video dimensions (700×576 pixels) and 6% of the area of the original video.

Each video was edited to have a 320 ms fade-in and fade-out. All videos were produced without any corresponding audio information, as the task was to focus solely on visual cues of interaction. Rating data for the video excerpts were collected via an online experiment, which was hosted in the Qualtrics online survey platform, with videos hosted on YouTube.

#### 3.2.2. Participants

In total, 26 volunteers (*M*_*age*_=38.7 years, *s*.*d*.=14.3, *age* *range*=23–70, eight males) completed the experiment. These participants were an opportunity sample recruited via social media. Participants had 9.08 years of musical training on average (*s*.*d*.=7.58, *range*=0–30 years); most classified themselves as serious amateur musicians (*N*=6), amateur musicians (*N*=8) or music-loving non-musicians (*N*=7).

#### 3.2.3. Procedure

Participants rated either the full video versions or the cropped video versions of the excerpts in terms of interaction between the two musicians. Interaction was defined as the degree of correspondence or coherence between the movement patterns of the musicians, similarly to the definition that had been provided to the annotators in the main study, and was rated on a 7-point scale (1 = low interaction, 7 = high interaction). The experiment lasted 28 min on average.

### 3.3. Results

The mean ratings of co-performer interaction for the four stimulus categories are listed in table 4. Bonferroni-corrected, paired-samples *t*-tests revealed that interaction ratings were significantly higher for true positives than true negatives (*p*<0.001), false positives (*p*<0.001) and false negatives (*p*=0.021). In addition, mean ratings of interaction for false negatives were significantly higher than false positives (*p*=0.003), suggesting ratings of the participants were more in line with the human annotators than the computational methods.

**Table 4.** Mean ratings of co-performer interaction by stimulus category.

	annotators	model	mean
stimulus	coded	predicted	interaction
category	interaction?	interaction?	rating (s.d.)
true positive	YES	YES	4.51 (0.91)
false negative	YES	NO	4.11 (0.94)
true negative	NO	NO	3.75 (1.00)
false positive	NO	YES	3.63 (0.97)

The primary aim of the analysis was to compare the degree to which the ratings from participants in Experiment 2 corresponded to the annotator’s and computational model’s categorizations of the video excerpts from Experiment 1. For comparison to the annotators, participant ratings for the true positive and false negative stimuli (cases in which the annotators identified interaction) were compared to ratings for the true negatives and false positives (cases in which the annotators identified non-interaction). We then tested the effects of these two aggregated stimulus categories, dataset (pulsed/non-pulsed duos) and visual information (cropped/full videos) on interaction ratings in a 3-way mixed ANOVA. No significant effect of visual information was found (*F*_1,24_=2.98, *p*=0.097), indicating that the full videos that had been seen by the annotators and the cropped versions that matched the ROIs from the OF analysis provided generally equivalent information about the level of interaction in a duo. A main effect of stimulus category (*F*_1,24_=49.53, *p*<0.001) indicated that ratings of co-performer interaction were significantly higher for the true positives/false negatives category (*M*=4.31) than for true negatives/false positives (*M*=3.69). A significant effect of dataset (*F*_1,24_=39.51, *p*<0.001) revealed that interaction ratings were higher for the pulsed (*M*=4.36) than non-pulsed duos (*M*=3.70). The pattern of mean interaction ratings by stimulus category was similar between the pulsed and non-pulsed duos; mean interaction ratings for the true positives/false negatives category were higher in both datasets (pulsed: *M*=4.76; non-pulsed: *M*=3.93) than mean ratings in the true negatives/false positives category (pulsed: *M*=3.96; non-pulsed: *M*=3.47). However, a significant interaction between dataset and stimulus category did emerge (*F*_1,24_=7.52, *p*=0.011), most probably explained by the greater difference in mean interaction ratings between the two stimulus categories for the pulsed duos (*M*_*difference*_=0.80) than the non-pulsed duos (*M*_*difference*_=0.46). No other significant interactions between predictors in the ANOVA were found.

For comparison to the computational model from Experiment 1, ratings for the true positives and false positives (cases in which the model identified interaction) were compared to ratings for the true negatives and false negatives (cases in which the model identified non-interaction). An ANOVA was run with these new stimulus categories, dataset and visual information included as predictors. Although co-performer interaction ratings for the true positives/false positives category were higher on average (*M*=4.07) than the ratings for the true negatives/false negatives category (*M*=3.93), no significant effect of the aggregated stimulus categories was found (*F*_1,24_=1.40, *p*=0.248). A significant interaction (*F*_1,24_=51.13, *p*<0.001) between stimulus category and dataset was also present. In the non-pulsed duos, mean ratings were higher for the true positives/false positives category (*M*=4.01) than the true negatives/false negatives category (*M*=3.43), but the opposite pattern of results emerged in the pulsed duos dataset (true positives/false positives: *M*=4.13; true negatives/false negatives: *M*=4.63). No other interactions between stimulus category, dataset and visual information were found in this analysis. Finally, the correlation was computed between the model predictions from the 1-predictor model from Experiment 1^4^ and the average ratings from the participants in Experiment 2 for each video stimulus. The correlation across the full dataset was not statistically significant, *r*_94_=0.172, *p*=0.093. However, there was a significant correlation between participant ratings and the model predictions for the non-pulsed duos dataset, *r*_50_=0.320, *p*=0.021, but not for the pulsed duos, *r*_42_=−0.192, *p*=0.212.

### 3.4. Discussion

The results of Experiment 2 provide validation and further elucidation of several aspects of Experiment 1. First, the cases in which both the annotators and computational model had classified an excerpt of a musical performance as a bout of interaction in Experiment 1 (true positives) were rated significantly higher in co-performer interaction by naive participants than all other stimulus categories. The ANOVA results, as well as the pairwise comparison of co-performer interaction ratings between false negatives and false positives, indicate that the interaction ratings given by participants in Experiment 2 were significantly aligned with the coded bouts of interaction/non-interaction as identified by the human annotators from Experiment 1, but were less aligned with the predictions generated by the computational model, particularly for the pulsed duos. A significant, positive correlation between the model predictions from Experiment 1 and participant ratings from Experiment 2 emerged only for the non-pulsed duos dataset, which corresponds with the fact that the 1-predictor model performed significantly more accurately in predicting human ratings of musical interaction for the non-pulsed duos than the pulsed duos in Experiment 1. Overall, these results provide affirmation for the annotation methods employed in Experiment 1 but also suggest that there may be certain visual cues (e.g. eye contact, direction of the movement) that are picked up as interactive by human raters to which the computational methods used here are not sensitive.

The absence of a significant effect or any interactions related to the ‘visual information’ variable indicates that the participants who were shown the cropped versions of the video did not perform in a noticeably different way from those provided with the full video. This result offers some evidence that discrepancies that arose between the annotators and computational models in Experiment 1 were not due to the different amount of visual information afforded to each. This is perhaps not entirely surprising, given that the annotators reported that their primary focus in coding bouts of interaction was on head and upper body movements (as coded in 97% of bouts), but rather provides additional support for this assumption.

A somewhat unexpected result was that pulsed duos were consistently rated higher in co-performer interaction than non-pulsed duos. One potential explanation for this is that the performers in the pulsed duo videos were very often nodding/moving along to the beat of the music throughout the course of a performance. When the annotators in Experiment 1 viewed the entire video recordings they often did not annotate such ‘beat-marking’ movement as interaction, as this would result in some cases in entire performances being classified as bouts of interaction. As the participants in Experiment 2 were only exposed to 7.5 s of each video recording, this type of contextual information that was taken into account by the annotators was not necessarily available. As such, movements that were directly influenced by auditory aspects of the performance (e.g. the musical beat) may have served as more salient cues to the Experiment 2 participants. In addition, the musical expertise of the Experiment 2 participant sample varied quite widely compared to the expert musician annotators from Experiment 1; thus, this more musically heterogeneous group may have been less precise in differentiating between communicative, ancillary movements and sound-producing movement or movements that serve other purposes.

To summarize, the results of Experiment 2 provide validation of several of the methodological decisions made in Experiment 1, and also highlight some areas for improvement in terms of developing computational tools that can approximate human judgements of interaction between musical co-performers.

## 4. General discussion

### 4.1. Summary of results

Human interaction involves the coordinated temporal exchange of information. Here we have investigated how interactions are coordinated in music performance, a domain which provides a particularly interesting object of analysis due to its diversity of temporal patterns and their hierarchical nature. In music, the ancillary movements of performers are assumed to relate to the coordination of phrasing and expressive intentions [34,82] in an analogous fashion to how turn-taking gestures facilitate interactions in conversation [10]. However, the existence of a shared temporal framework in music (e.g. pulse, metre, phrase structures) and the alternation between different modes of playing together (from soloist/accompanist roles to joint, synchronized or interlocking playing) make such intentions putatively more complex to execute. Another important aspect of interactions in music performance is their multimodal nature; musical interactions might manifest themselves differently in auditory and visual channels, providing complementary information. Specifically, ancillary movements are assumed to provide visual communicative signals that disambiguate, reinforce or augment auditory information related to musical structure and expression [35,73,83]. This is analogous to how head movements and upper body and hand gestures have an important role in the perception of speech [84], including directing attention [85] and signalling turn-taking in conversation [5]. In the present work, we assumed that the recurrent ancillary oscillations of the upper bodies of musical performers are essential coordination cues in improvised performances, where there is no score or conductor to guide the interaction.

In Experiment 1, we contrasted two styles of improvized music to explore how interactions are visually coordinated in music. One dataset comprised performances relying on a steady pulse and one set avoided such a pulsed framework, providing a natural variation in the level of temporal regularity they contained. Visual interactions (as coded by expert musician annotators) in both datasets were generally related to joint performer movements, but the strength of this association varied according to the metrical regularity of the music. In non-pulsed music, visually apparent co-performer interactions were adequately predicted from the energy of the CWT of the movements of the two performers over a broad frequency range. In pulsed music, however, such a broad model failed to significantly account for the interactions. Only when a more complex model was specified, with energy calculated over specific frequency bands of CWTs nested with other predictors within a tree model, could a reasonable degree of visual interactions between the performers be predicted. This asymmetry of the success of the movement cues to predict interactions is consistent with the notion that visual cues provide complementary information in communication [1,73,83,86]; in pulsed music, co-performer coordination may be mainly achieved by tracking of well-established structures in the music (e.g. the regular pulse, phrases and chord changes which form the foundational structure in standard jazz) in the auditory channel, leaving less influence for ancillary movements and visual information. These results bear conceptual similarity to those of Moran *et al.* [59], who investigated communicative behaviours using a subset of the present data and found that participants were more successful at distinguishing real from fake pairings of the musicians in the non-pulsed than the pulsed duos based on visual back-channelling cues from the non-soloing duo member. Whether the ancillary movements and gestures in pulsed jazz performances are meant to be communicative or could just be visible traces of how the musicians embody the structures in music is a question to which this study cannot provide direct answers. However, the success of the movement cues in predicting visually apparent interactions in the non-pulsed duos suggests that they may have a pronounced role at least in music which otherwise does not contain strong auditory coordination cues. When considered in the light of theoretical views concerning the relationship between interpersonal coordination at the level of co-performers’ sounds versus ancillary body motion [23], our results imply that the role played by these complementary sources of information may change depending on the temporal regularity of the music. Specifically, ancillary motion may become especially relevant to co-performer communication in music that lacks a regular pulse-based metric hierarchy that can be used to generate predictions over multiple timescales [41,87–89].

It is remarkable that here the simplest model consisting only of the broadband CWT energy could achieve a classification accuracy rate of above 0.80 for interactions in non-pulsed music, and the other predictors (audio features, movement quantity, etc.) did not make significant individual contributions to the model. Nevertheless, the low accuracy rate of the same model in predicting interactions in pulsed duo performances underscores the fact that both the cues for coordinating interactions and the importance of visual information in co-performer communication may vary drastically across musical styles. The ease with which the joint periodic movements across multiple timescales could predict interactions in non-pulsed music is perhaps unsurprising, because a hierarchical metrical structure is largely absent in such traditions and the performers cannot rely on predictable temporal patterns (beat, metre, bar, phrases) to coordinate their performance. Instead, as in conversation, they provide gestural cues by swaying and nodding simultaneously, which typically use similar periods of movement. In pulsed duos, where the performers were following a loose musical script consisting of sections of joint performance separated by sequences of alternating solo sections that adhered to a metrical hierarchy, the ancillary movements were not particularly indicative of the interactions. Some of the annotated bouts of interaction could, however, be captured by a more complex account of the frequency ranges over which the joint movements were taking place. The reliance on more specific frequency bands (e.g. the band centred around 0.4 Hz) rather than the broadband CWT predictor in the pulsed duos may be due to the quite narrow musical tempo range (120–150 beats per minute) implicated in these performances of the same jazz standard, which could have afforded less diversity in terms of movement periods of the performers.

### 4.2. Limitations and future directions

The manual annotation method implicated in Experiment 1 has its drawbacks. It is a subjective task, requiring a careful operational definition of the concept of musical interaction, which will nevertheless be subject to different interpretations by different coders. It is also challenging to identify the precise onsets and offsets of the bouts of interactions, because interactions evolve over time and different coders may set different subjective thresholds for the amount of evidence that needs to be accrued before a bout can be identified as an interaction. In addition, when working from a single video recording, the perspective of the annotator is necessarily different from that of the individual performers involved in the musical interaction; in this particular case, the annotators were able to see the face of one performer more clearly than the other in each duo (due to the camera angle) and thus may have missed certain visual cues in terms of eye contact or facial expressions. Despite these challenges, we obtained high measures of interrater agreement between coders that were validated by ratings from an independent sample of participants (Experiment 2). However, we acknowledge that the operational definition and coding method used here is just one possible approach that could be expanded upon in future to include self-reports and interviews with the musicians themselves (cf. [90]), explorations of the intentional nature of interactions, comparisons of successful/unsuccessful attempts at interaction, directional interactions (e.g. leadership roles), etc.

It is probable that the movement cues that we focused on (periodic relationships between the two performers’ movements) are not sufficiently nuanced to pick up all visual interactions that were present in these performances. Although we explored the contributions of various additional predictors, several of these—such as the amplitude of the movements, measures of the musical qualities of the performances (dynamics, pulse clarity) and supplementary visual information (Experiment 2)—did not provide substantial further information in terms of identifying bouts of interaction. However, there are other, more subtle ways musicians could signal important events during a performance, such as by making unique, single gestures or eye contact, neither of which can be detected by the current approach. Series of studies have documented the different ways musicians coordinate their actions in duo performances with gestures and looking behaviours [39,75,83]. For instance, Davidson [75] described qualitatively how one duo used body sway and head nods to generate direction in musical phrases, while another duo used circling movements to indicate the end of phrases. In the present study, we did not capture the gestural properties of the movements, but it is plausible that a more precise vocabulary of gestures could be present in the broad movements that were tracked. Such communicative functions (affect displays, regulation or emblems) have been suggested to constitute a separate vocabulary in music [91], but the specifics of such patterns remain to be explored across traditions, performances and instruments.

The majority (97%) of the ancillary movements that were coded as interactive by the annotators in Experiment 1 comprised the performers’ upper bodies—specifically, head and torso movements. The focus on upper body cues was further reinforced by the results of Experiment 2, which indicated that cropped videos containing only the upper bodies of the performers provided enough information for raters to discriminate interactive from non-interactive bouts. However, future studies should investigate whether such a finding is transferrable to other musical styles and instruments. For instance, singers have been noted to make use of hand gestures [92], which did not feature as a prominent communicative cue in the present study of instrumental musicians.

There are a host of other possible factors that might influence the frequencies of the movements produced by each performer. For instance, standing and sitting postures have different points of balance for movements, which is assumed to influence movement type and periodicity [93,94]. Also, the physical layout and visibility of co-performers afforded by different instruments can result in different movement patterns [29]. Akin to conversation, it is plausible that the cues harnessed in coordinating musical performance are flexible and will reflect the most salient and accessible types of communication devices available [95].

In terms of the methodological choices, wavelet analysis proved to be a flexible tool for capturing time–frequency patterns from continuous movement data. Although WTs are routinely used in signal processing and in certain areas of behavioural sciences [67], they have not previously been applied in studies of interaction in music, except for demonstration and visualization purposes [24]. One way to expand this approach would be to extract additional information from the wavelet transformations, such as coherence, which is the cross-correlation of the two wavelet spectra. One could also test other analysis techniques such as CRQ. Cross-recurrence analysis emphasizes the detection of recurrent temporal patterns, which could be useful in research on automatic discovery of the turn-taking patterns or structural elements of the music.

The notion that coordination takes place at different timescales was partially explored in the present study. The specific frequency bands of the CWT allowed us to explore a diverse range of ancillary movements, from rapid head nods to slow body sway and everything in between. Studying the synchronization of note onsets between the performers would be a natural extension of the study, to link the factors contributing to musical synchronization with the cues used to coordinate interaction [23]. For instance, Bishop & Goebl [96] have found that note-level synchrony is related to kinematic features of communicative head gestures. Such a shift to a lower temporal level (e.g. note-to-note synchrony) could also be useful in harnessing the phase information used in the present study more appropriately; phase is only relevant when the joint frequencies are closely matched and the level of synchronization is high [97]. It is also worth noting that the performers’ movements were extracted in the present study from standard video streams (25 Hz) with computer vision tools [51]. Although such information lacks sufficient temporal resolution for fine-grained synchronization analysis, it does provide a wealth of information for behaviours above the level of the musical beat and can pick up on most ancillary movements, which typically occur over longer timescales than sound-producing movements (e.g. of the order of seconds [38,73]).

The methodology adopted here allows an effortless expansion into other musical genres, or even ensemble performances from different cultures that have been documented on video. The emphasis on visual coordination cues in non-pulsed music that was revealed in the present study suggests that this approach might be particularly fruitful for studying other music that lacks a regular temporal structure, such as the works of numerous contemporary composers from Boulez [98] to Nancarrow [99], the opening sequences (alap) in north Indian classical music ([[100], pp. 95–103; [101]]) or in unmetred patetan sections in Javanese gamelan music [102]. In addition, a logical follow-up to the descriptions we have provided of potentially communicative visual cues in this paper would be to investigate causal relationships by manipulating such cues directly; for instance one could test how the coordination and quality of periodic movement patterns are affected by the elimination of visual or auditory feedback received by co-performers across different performance conditions, or how masking certain portions of the visual information might affect co-performer coordination more dramatically than others (e.g. [103]).

### 4.3. Conclusion

The present work introduced a novel method for quantifying visually observed co-performer interaction in music ensemble performances using measures of the shared periodic movements of performers across multiple timescales. Statistical models incorporating these quantitative measures were successfully able to classify manually annotated bouts of visual co-performer interaction/non-interaction in improvising duos, as coded by expert musicians. The models were particularly effective in predicting such interactions in non-pulsed improvisations, which highlights the importance of the visual modality for coordinating ensemble performances in cases where the musicians cannot rely on a regular, predictable structure in the auditory domain. Models of interaction in pulsed music required a more complex combination of predictors, which highlights some discrepancies in the use of the visual communication channel for pulsed versus non-pulsed music and suggests that the narrow tempo range over which the pulsed performances were executed may have constrained the diversity of movement periodicities. The methods developed here can be easily transferred and extended for use with other musical styles, to explore potential commonalities and divergences in the types of movement cues and coordination that are used across different musical ensemble types and traditions.

## Supplementary Material

Predictor Contributions of the Tree Models

## Footnotes

1 Though see [52] for some caveats to using cross-correlation for time-series analysis.

2 http://www.infomus.org/eyesweb_ita.php.

3 These frequencies correspond to movement periods of 2.9, 2.3, 1.7, 1.1 and 0.5 s. The range of each band was determined as the movement period ±0.3 s.

4 This variable was log-transformed due to a positively skewed distribution.

## Data accessibility

The original data were reported in a study by Moran *et al.* [59]. The stimuli (short point-light displays) for that report are available at http://dx.doi.org/10.7488/ds/251. Experiment 1 videos are available at http://datashare.is.ed.ac.uk/handle/10283/2840, which is part of the Edinburgh data repository. All data used in Experiments 1 and 2 are available at UK Data Service ReShare at https://dx.doi.org/10.5255/UKDA-SN-852847.
