# Supplementary material for: Shared periodic performer movements coordinate interactions in duo improvisations
Source: R Soc Open Sci. 2018 Feb 21;5(2):171520. doi: 10.1098/rsos.171520 (PMC5830756; doi:10.1098/rsos.171520)
Supplement: Predictor Contributions of the Tree Models [file rsos171520supp1.pdf]

Electronic Supplementary Materials 1

Predictor Contributions of the Tree Models

To probe the interactions between the best performing predictors, we visualised these relationships in individual tree models separately for the Pulsed and Non-pulsed Duos. For simplicity in visualising and interpreting the potentially complex interactions between variables, we included three predictors in each of the tree models. These 3-predictor tree models successfully predicted bouts of interaction in both datasets (AUC = 0.779 in the Non-pulsed Duos, AUC = 0.745 in the Pulsed Duos, using the training dataset for model building and evaluation subsets for prediction).

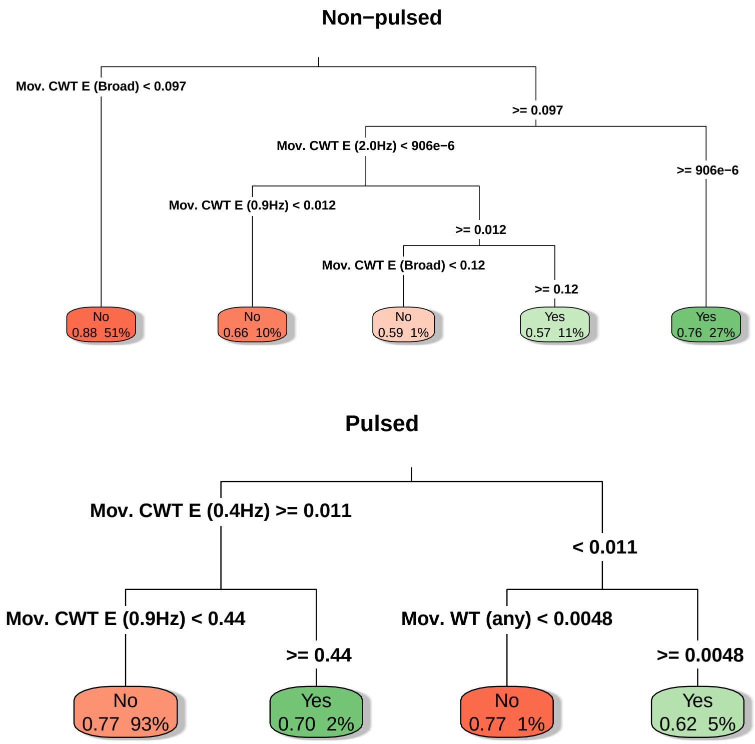

Figure 1. Examples of pruned decision trees using 3 predictors for both datasets. The decision label "No" refers to "No interaction" and "Yes" to "Interaction". The first number below the decision label indicates the probability of the second class and the second number the percentage of the amount of observations at the end state.

In the Non-pulsed Duos, bouts of interaction are first characterised by high *Movement CWT Energy (Broad)* values, as values over 0.097 suggest interaction, whereas low values (< 0.097) lead to classification as non-interaction. Elevated levels in a shared periodic movement in a specific frequency ranges (2.0 and 0.9Hz) also signify interaction between performers. In the Pulsed Duo performances, the decision splits rely on shared movements in more specific CWT Energy bands. High values of *Movement CWT Energy (0.4 Hz)* most likely indicate interaction, unless there is considerable amount of co-occurring periodic movement at 0.9 Hz frequency as well. Also, if the *Movement WT Energy (any)* is elevated, the bouts are probably interactive.
